# Stunting Among HIV-Exposed and HIV-Free Children in eSwatini: A Retrospective Evaluation of Associations with Birthweight, Feeding, and Caregiving Practices

**DOI:** 10.3390/nu18020198

**Published:** 2026-01-08

**Authors:** Bareng A. S. Nonyane, Letha Varughese, Jigna M. Dharod, Xolisile Dlamini, Andrea Ruff, Maureen M. Black

**Affiliations:** 1 Bloomberg School of Public Health, Johns Hopkins University, 615 N. Wolfe Street, Baltimore, MD 21205, USA; lethapinto@gmail.com (L.V.); aruff1@jhu.edu (A.R.); 2 Department of Nutrition, School of Health and Human Sciences, University of North Carolina Greensboro, Greensboro, NC 27412, USA; jmdharod@uncg.edu; 3 Ministry of Health, Mbabane H100, Eswatini; 4 Department of Pediatrics, University of Maryland School of Medicine, Baltimore, MD 21201, USA; mblack@som.umaryland.edu

**Keywords:** HIV-exposed, HIV-free, stunting, social determinants of health

## Abstract

**Background/Objectives**. Associations between stunting and dietary practices have been understudied among HIV-exposed and HIV-free children. We investigated associations between birthweight, socio-demographics, and dietary and feeding practices with stunting at 9 and 18 months among children in eSwatini. **Methods**. We used generalized linear mixed models (GLMs) with a logit link to characterize associations between stunting and birthweight quartiles, socio-demographics, maternal characteristics, and infant dietary diversity scores. We examined the moderating effects of dietary factors on relations between birthweight and stunting. Generalized structural equation models characterized direct and indirect associations between exposures and stunting at 18 months, mediated through stunting at 9 months. **Results**. We included 367 HIV-exposed and HIV-free children. Infants in the third and fourth birthweight quartiles had reduced odds of stunting at 9 months [adjusted odds ratio (adj OR) 0.24 (IQR 0.11, 0.55), *p* < 0.001; 0.10 (0.03, 0.33), *p* < 0.001, respectively]. Moderation by dietary diversity was limited to a relative decline in the second birthweight quartile. Stunting prevalence significantly increased from 9 months (21%) to 18 months (37%). Mediated by stunting at 9 months, there were significant direct and indirect effects of birthweight and dietary diversity on stunting at 18 months. **Conclusions**. Among HIV-exposed and HIV-free children, stunting prevalence increased with age and was associated with lower birthweight. Dietary diversity attenuated stunting risk among children in the second birthweight quartile. Prenatal strategies to reduce low birth weight (LBW) and additional attention to the social determinants of health, particularly dietary diversity, are warranted in programs and policies to reduce stunting.

## 1. Introduction

Increases in antiretroviral therapy (ART) among pregnant women living with HIV and infant ART prophylaxis have reduced vertical transmission of HIV infections among young children, with associated increases in the number of HIV-exposed and HIV-free children [1]. Based on the 2023 UNAIDS report, approximately 16 million children are classified as HIV-exposed and HIV-free globally, with 70% in eastern and southern Africa [2]. HIV-exposed and HIV-free children experience increased risks of morbidity and mortality, preterm birth, growth faltering, and low motor and cognitive scores compared with unexposed children [3,4,5]. Modifications to the intrauterine environment due to HIV infection, such as endometrial, placental, and amniotic infections, are associated with fetal growth restriction and low birth weight (LBW) [6]. Although HIV-exposed and HIV-free children may be uniquely vulnerable to stunting, evidence is mixed, with some studies finding increased risk [7] and others finding no differences compared to HIV-unexposed and uninfected (HUU) children [8]. The differences in findings may be related to the variability in considering comorbidity, such as infections, or environmental variables, such as poverty or food insecurity, that may also increase the risk for stunting.

Stunting, often considered an indicator of chronic undernutrition, increases children’s risk for morbidity and poor academic performance. The consequences of stunting can extend into adulthood, with adverse health conditions and negative associations with human capital, including economic productivity [9]. Nutrition interventions have had limited impact on stunting, particularly among populations at risk for infections [10]. Recent evidence has found associations between stunting and non-nutritional factors, including poverty and low maternal education [11,12].

The quality of children’s care and feeding has consistently been positively related to children’s growth and development [13,14], including HIV-exposed and HIV-free children [15,16]. The complementary feeding phase (ages 6–24 months) is a particularly vulnerable period for children’s health. Not only do children’s inherited antibodies decline, increasing their risk for infections, they are also transitioning from breast milk (or formula) to semi-solid foods, exposing them to new foods and feeding practices, and their fine and gross motor skills mature, increasing the likelihood of putting objects into their mouth. Recommendations are to provide a diet diverse in energy, protein, and micronutrients and to engage in responsive feeding practices [17]. However, the association between feeding practices, such as dietary diversity and responsive feeding, and stunting have been understudied during the complementary feeding phase among HIV-exposed/HIV-free children, creating a knowledge gap.

The Nurturing Care Framework, endorsed by the World Health Assembly in 2018, provides a roadmap of the care and experiences that young children need for healthy growth and development [18]. The Framework builds on the Bioecological Model [19] by emphasizing that children’s development is shaped by bidirectional interactions with the environment, including caregivers. Children’s access to adequate nutrition and healthcare, protection from household and external threats, and opportunities for learning and responsive caregiving within a stable and nurturing home are interconnecting components of Nurturing Care [18,20].

Using the Nurturing Care Framework as a conceptual base, we aimed to investigate the associations between infant birth characteristics, household socio-demographic factors, and infant dietary and feeding practices with children’s stunting. We selected two timepoints within the complementary feeding period: 9 months represented children’s early adjustment to consuming foods and 18 months represented children’s adjustment to a greater variety of foods and, in many cases, partially feeding themselves.

We used data from a study conducted in eSwatini, a country in southern Africa with an estimated HIV prevalence of 24% of the population—among the highest in the world [21]. Women have a higher prevalence (32%) than men (16%), and approximately 32% of children under age 14 years are HIV-exposed and HIV-free [1]. Although eSwatini has reduced the number of annual new HIV infections by 70% since 2010 and reached the 95-95-95 targets by the end of 2024, there are still large numbers of children being born to women living with HIV [22]. The prevalence of stunting is approximately 25% among children under 5 years, and approximately 35% for children under 2 years [23]. Thus, eSwatini represents a high-risk environment for young children.

**Figure 1:**
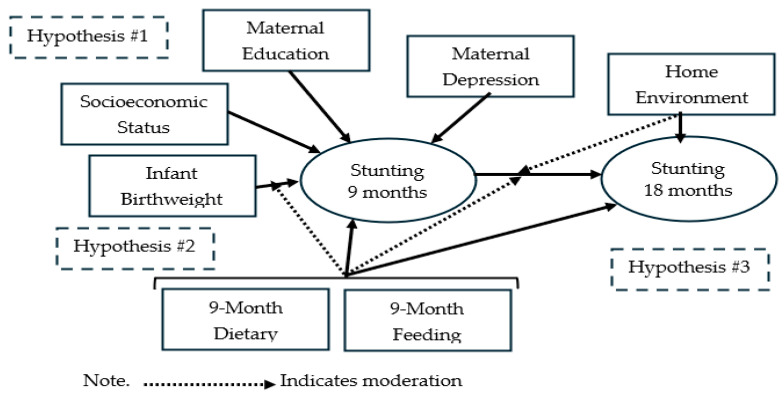
Conceptual framework of stunting risk and hypotheses.

We tested three hypotheses (see Figure 1):

**Hypothesis** **1.** *Stunting at 9 months is associated with infant birthweight, socio-demographics, maternal characteristics, and infant dietary and feeding practices*.

**Hypothesis** **2.** *The association between stunting at 9 months and infant birthweight is moderated by infant dietary and feeding practices, such that the association is attenuated by positive dietary and feeding practices*.

**Hypothesis** **3.** *Stunting at 9 months mediates the association between birthweight and stunting at 18 months. As a corollary, we hypothesized that positive infant dietary, feeding, and caregiving practices moderate the association between stunting at 9 and 18 months such that healthier practices attenuate the effect on stunting*.

## 2. Materials and Methods

This observational exploratory study used data from a cluster randomized trial conducted in eSwatini between January 2016 and May 2018 to evaluate the impact of a Nurturing Care-based intervention integrated into antenatal care/prevention of vertical transmission (ANC/PVT) programs on children’s neurodevelopment [24]. Recruitment methods have been reported elsewhere [24] and are summarized here. Six ANC/PVT clinics that were supported by the mothers2mothers nonprofit organization served as intervention clinics, and participants received home, clinic, and community interventions focused on early childhood development. Nine clinics that were supported by PEPFAR partners served as the comparison. They provided care as usual, with no intervention from mothers2mothers.

### 2.1. Participants

We enrolled women in their third trimester of pregnancy who were confirmed HIV-positive and intended to remain in the clinic catchment area for 18 months. Details of the participant flow diagram have been published previously (Figure 1) [24]. Briefly, 429 children were born alive to mothers living with HIV attending antenatal care at the study facilities. Of these, 368 (188 intervention, 180 comparison) had a 9-month assessment, and 346 (180 intervention, 166 comparison) had an 18-month assessment. For this secondary analysis, 367 (187 intervention and 180 comparison) had the stunting measurement at 9 months, and 339 (177 intervention and 162 comparison) had the stunting measurement at 18 months. Missing stunting outcome (in 8 infants) was found to be unrelated to any of the participant characteristics.

Ethical approval was granted by the Institutional Review Board at the Johns Hopkins Bloomberg School of Public Health # 00006298, 21 December 2015, and the eSwatini National Health Research Review Board. All participants provided written consent for themselves and their infant.

### 2.2. Procedures

All data collectors were trained to administer the measures, including anthropometry, and to collect the necessary information from clinic charts. Data collectors were supervised with regular attention to quality control. Most data were collected on tablets with Magpi software.

We collected demographic information and baseline data on maternal characteristics at enrollment. At ages 9 and 18 months, we collected children’s weight, length, and dietary and feeding measures. At 12–15 months, we conducted home visit observations. We timed the visits to align with the children’s feeding and napping schedule. Over 90% of children received a DNA PCR test.

#### 2.2.1. Growth Outcomes

Data collectors used standard procedures to measure weight (kg) and length (cm) in triplicate using a digital scale and a stadiometer with regularly scheduled calibration procedures. We defined growth measurements according to the World Health Organization (WHO) standard z-scores. Weight-for-age, weight-for-length, and length-for-age z-scores <= −2 were considered underweight, wasting, and stunting, respectively [25]. We created binary outcomes for analysis, and our analysis focused on stunting.

#### 2.2.2. Infant Characteristics and Socio-Demographics

We gathered infants’ birthweight (kg) from hospital records. We divided the data into quartiles for analysis based on the distribution of the data (1.25–2.9, 2.95–3.1, 3.15–3.5, 3.57–5.0). We conducted a baseline interview of socio-demographic characteristics, including caregivers’ age, education, employment, marital status, assets, housing, and income-expenditure information.

#### 2.2.3. Infant and Young Child Feeding (IYCF)

We used the WHO-developed IYCF feeding definition and methods for measurement and analysis [26]. We calculated children’s minimum dietary diversity, defined as consumption of foods from at least 5 of the 8 food groups in the previous 24 h. We generated 3 categories of the diversity score based on the distribution of the data: <5 (does not meet criteria), =5 (just meets criteria), and >5 (exceeds criteria). Minimum meal frequency, defined as eating at least 2 meals/day for breastfed children aged 6–8 months, at least 3 meals/day for breastfed children aged 9–23 months, and at least 4 meals and 2 milk feeds/day for non-breastfed children 6–23 months.

#### 2.2.4. Responsive Feeding

From a 19-item responsive feeding questionnaire [27], we identified items that represented responsive (recognizes when child indicates fullness), controlling (pressuring child to eat), and indulgent (giving food throughout the day without a schedule) feeding. The items were scored on a 4-point Likert scale based on frequency (1—almost never, 2—occasionally, 3—most days, 4—almost every day). We stratified the responses for each category into quartiles. Higher values and higher quartiles indicate more frequent endorsement of the feeding practice. The quartiles were converted to bivariate codes (1—fourth quartile, meets criteria; 0—quartiles 1–3, does not meet criteria).

#### 2.2.5. Maternal Mental Health

We measured maternal depressive symptoms using the Edinburgh Postnatal Depression Scale. We defined scores => 13 as depression, following findings from a study in South Africa that demonstrated acceptable sensitivity and specificity using this cut-off point [28].

#### 2.2.6. Household Characteristics and Caregiving Practices

After pilot testing, we selected 16 items from the infant-toddler version of the Home Observation for the Measurement of the Environment (HOME) inventory that were culturally appropriate for eSwatini homes [29]. Assessments were done at the 12- to 15-month postnatal home visit by data collectors who were unaware of participants’ intervention arm. Scores were summed, with high scores indicating more responsive mother–child interactions.

### 2.3. Sample Size

The sample size for this secondary analysis was fixed at the number of mother–infant pairs required to meet the objective of the main study. This was 15 to 27 per clinic to demonstrate a 7.5-point difference in Mullen scores of neurodevelopment between study arms.

### 2.4. Statistical Analyses

We used descriptive statistics to summarize household and maternal characteristics and infant birth characteristics stratified by stunting status at 9 months and 18 months. Chi-squared tests were used to evaluate the association between these characteristics and stunting status (stunted vs. not stunted) at 9 and 18 months.

Generalized linear mixed models (GLMs) and generalized structural equation models with a logit link were used to test the hypotheses. These were adjusted for study arms from the original trial [24] and used robust variance estimation to account for clinic clustering effects. For Hypothesis 1, we evaluated the association between stunting at 9 months and the exposures [birthweight, socio-demographic characteristics, maternal depression, dietary diversity score, minimum meal score, responsive feeding, and controlling feeding]. We evaluated potential collinearity among all exposures and found a variance inflation factor less than 1.5, indicating no evidence of strong correlations among exposures. Potential exposures and confounders were considered based on prior literature. Each variable was individually evaluated for association with stunting using chi-squared tests. To achieve parsimonious models, variables with *p*-value < 0.2 in the chi-squared tests were included in the multivariable regression alongside study arm, child sex, and exact age, a priori confounders. The socioeconomic status (SES) variable was derived using principal component analysis combining household asset ownership. In response to missing values, we also used availability of a toilet, electricity, and clean running water as proxy variables that indicate SES and have been shown to have associations with child growth. Birthweight was divided into quartiles.

For Hypothesis 2, we followed a similar approach of building a multivariable model as for Hypothesis 1, and the model was extended to include the interaction term between birthweight categories and dietary diversity score categories at 9 months.

Hypothesis 3 was tested using a generalized structural equation model (GSEM) that characterized two hypothesized paths. The first was a direct association (model path 1) between stunting at 18 months and the exposures of stunting at 9 months, birthweight, dietary diversity score, and home caregiving observation score. The second was an indirect association (model path 2) that links birthweight, dietary diversity score, and other potential confounders to stunting at 9 months, which is further linked to stunting at 18 months. Parameter estimates were derived through the default adaptive Gauss–Hermite quadrature estimation in Stata software version 16. Robust variance estimation was used to account for clustering by clinic site. We also tested the moderating effects of home caregiving practices on birthweight. The feeding practices and dietary measures used in this analysis were collected at the 9-month timepoint because they were not available at 18 months.

## 3. Results

### 3.1. Sample Characteristics and Attrition

We included 367 of the 368 children who participated in the 9-month evaluation (one did not have data on stunting) [24]. At 18 months, retention was 92% with 339 children participating. Characteristics of the infants, mothers, and households, stratified by stunting status at both time points, are given in Table 1. Stunting prevalence was 20% (76/367) at 9 months and 36% (123/339) at 18 months. Two infants, one in each arm, tested positive for HIV. In the unadjusted tests, low birthweight and low SES were associated with stunting (Table 1, Supplemental Table S1). At 18 months, mothers’ lower education levels and dietary diversity scores below 5 were also significantly associated with stunting. Dietary diversity score at 9 months, minimal mean frequency, and the three feeding practices were not associated with stunting (Table 1).

Most potential predictors had few missing values (<5%). SES and initiation of ART had up to 10% missingness in the subgroups of Supplemental Table S1. Infant nevirapine use had up to 30% missingness in the sub-categories due to incomplete medical records. We did not conduct imputation as this is an exploratory analysis.

### 3.2. Regression Models for Hypothesis 1

The multivariable regression model results testing Hypothesis 1 (Table 2) indicate that the third and fourth birthweight quartiles were associated with reduced risk of stunting at 9 months compared to the lowest quartile, after adjusting for all other potential confounding factors [adjusted odds ratio (adjOR) 0.24 (IQR 0.11, 0.55), *p* < 0.001, 0.10 (0.03, 0.33), *p* < 0.001, respectively]. Compared to scores < 5, dietary diversity scores = 5 [adjOR 0.86 (0.44, 1.71), *p* = 0.68] and >5 [adjOR 0.47 (0.21, 1.08), *p* = 0.08] were not statistically significantly associated with stunting risk reduction.

### 3.3. Regression Models for Hypothesis 2—Interaction Analysis

**Figure 2:**
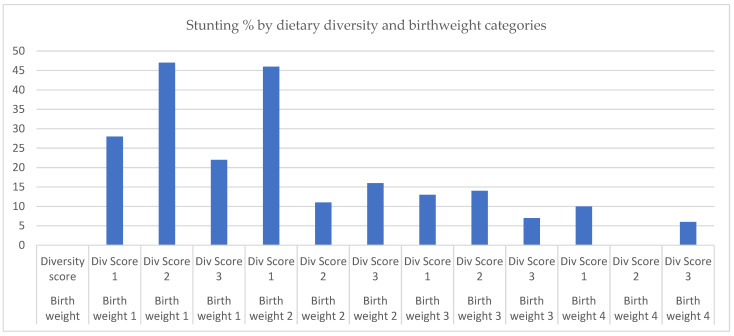
Stunting percentage by dietary diversity and birthweight categories. Note: Birthweight 1 = 1.25–2.9 kg, Birthweight 2 = 2.95–3.1 kg, Birthweight 3 = 3.15–3.5 kg, Birthweight 4 = 3.57–5.0 kg. Dietary Diversity Score 1 = <5, Dietary Diversity Score 2 = 5, Dietary Diversity Score 3 = >5.

To investigate Hypothesis 2, we extended the model for Hypothesis 1 to include an interaction term between birthweight categories and dietary diversity score categories. Figure 2 suggests that the main factor associated with increased risk of stunting is being in the lowest birthweight category, irrespective of the diversity score level. As shown in Table 3, the main effect of birthweight among low dietary diversity categories remains significantly associated with reduced risk of stunting. Relative to the lowest birthweight and dietary diversity categories, a combination of a higher birthweight (quartile 2) and higher diversity score was associated with a significant relative decline in stunting risk [interaction term [adjOR. 0.07 (0.01, 0.51), *p* = 0.01]. This finding was not observed in combinations of higher birthweight and diversity score categories (Table 3, Figure 2).

### 3.4. Regression Models for Hypothesis 3—Structural Equation Modeling

For Hypothesis 3, we evaluated the prevalence of stunting at 18 months with stunting status at 9 months as a risk factor. Of the total 76 participants who were stunted at 9 months, 63 (83%) were stunted at 18 months, while 6 (8%) were lost to follow-up. Of the 291 who were not stunted at 9 months, 59 (20%) were stunted by 18 months, and 28 (10%) were lost to follow-up. Thus, the losses to follow-up were similar between the two groups (8% vs. 10%). A generalized structural equation model characterized a two-part model, where the first path modeled a direct association between birthweight, home observation score of caregiving practices, and dietary diversity with the outcome of stunting at 18 months. The second modeled a path associating birthweight, household, and maternal characteristics with stunting at 9 months, which further led to stunting at 18 months (Table 4). This model showed significantly reduced odds of stunting at 9 months associated with the third and fourth birthweight categories. There was an increased odds of stunting at 18 months associated with stunting at 9 months [adjOR 23.80. (9.10, 62.26) *p* < 0.001]. The high odds ratio and wide confidence intervals reflect the notable strong impact of being stunted at an earlier age on the risk at a later age, even when the phenomenon is observed on a relatively small number of infants—the majority of those who were stunted at 9 months were also stunted at 18 months. The fourth birthweight category and dietary diversity score > 5 were associated with reduced odds of stunting [adjOR 0.34 (0.16, 0.75) *p* = 0.007; 0.41 (0.18, 0.91) *p* = 0.029, respectively]. Supplementary Table S2 shows the coefficients of the hypothesized total, direct and indirect effects of birthweight, and dietary diversity from this model shown in Table 4. This suggests that the highest level of diversity score has significant direct and indirect effects on stunting at 18 months (Supplementary Table S2). However, after extending this model to include a potential moderating effects of positive caregiving practices and dietary diversity scores, we found no evidence of a significant association. (Supplementary Table S3).

## 4. Discussion

In our study of stunting among HIV-exposed and HIV-free children in eSwatini, we found that higher birthweight was associated with reduced odds of stunting at 9 months and 18 months (Hypothesis 1). Secondly, we found suggestive evidence that the association between lower birthweight and stunting was altered by dietary diversity at 9 months among a subset of children (Hypothesis 2). Thirdly, there was a significant increase in the prevalence of stunting from 9 to 18 months and both direct and mediated effects of birthweight and dietary diversity on stunting at 18 months (Hypothesis 3). There was no evidence of a moderating effects of dietary diversity or home observation of caregiving practices on the association between birthweight and stunting at 18 months.

The first finding is consistent with evidence that lower birthweight increases the risk for stunting across multiple conditions [30]. Similar findings have been shown in a recent meta-analysis and systematic review [31]. LBW (defined as <2.5 kg) can signify restrict fetal growth and immature organ and physiological development, increasing the risk of mortality and morbidity, including stunting, among children under age 5, with a moderately higher risk in Asia than in Africa [21,32].

The second finding provided suggestive evidence that a small increase in birthweight (second quartile, 2.95–3.1 kg) plus dietary diversity was associated with reduced stunting prevalence at 9 months. These findings suggest that at the lowest levels of birthweight (first quartile), biological factors may limit linear growth, leading to stunting. However, as birthweight increases to the second quartile, biological constraints may be attenuated by dietary diversity. At higher levels of birthweight (third and fourth quartiles), there is no association with stunting, suggesting that postnatal factors relate to linear growth. The social determinants of health, conditions where people are born, grow, live, work, and age, along with access to power and money, including poverty and lack of access to healthcare, have been associated with multiple aspects of health [33]. Although some social determinants are fixed, such as birth place, others are modifiable, such as feeding practices. A Cochrane collaboration found that in the context of severe poverty, nutrition-specific interventions were not effective in reducing either LBW or stunting [34]. The collaboration recommended the investigation of a multisectoral strategy that included nutrition-specific interventions as well as “up-stream” programs and policies that addressed the social determinants of health and involved governmental, non-governmental, and community organizations.

The significant increase in the prevalence of stunting from 9 to 18 months is consistent with the stunting prevalence in the general eSwatini population, regardless of HIV exposure. During the 9- to 18-month period, infections often increase as children become more mobile, have poorly developed hygienic practices, and often interact with other children, particularly in childcare settings [35]. At 18 months, we found direct and mediated (by stunting at 9 months) effects of birthweight and dietary diversity score, such that compared to the lowest categories, children in the higher birthweight quartiles and higher diversity score had significantly reduced odds of stunting at both 9 and 18 months. These findings are encouraging because they suggest that a modifiable factor, such as dietary diversity, can attenuate the biological constraints of slightly lower birthweight, although not the biological constraints of LBW.

Caregiving practices, measured through observation at 12-15 months, were not associated with stunting at 18 months. One explanation may be that the biological constraints associated with lower birthweight on linear growth impacted stunting at 9 months, and the caregiving observation at 12–15 months did capture the conditions associated with stunting earlier in life. A recent systematic review found that complementary feeding practices and supplementary food were associated with increases in height-for-age z-scores and reductions in stunting [36]. However, a study among HUU children in Ghana found that although IYCF indicators were not associated with stunting in multivariate analyses, LBW and household variables, including low socio-economic status, male gender, and unimproved toilet facilities, were significantly associated with stunting [37]. These findings point to the need for the inclusion of both nutritional factors and the social determinants of health in investigations of stunting among HIV-exposed and HIV-free children as well as HUU children.

A recent systematic review and meta-analysis found that HIV-exposed and HIV-free children have more growth faltering (underweight and stunting) than HUU children through the first 24 months [38]. A study from Botswana found that that among HIV-exposed and HIV-free children under 12 months and greater than 24 months, the association with stunting was mediated by LBW [39]. In contrast, between 12 and 24 months, when weaning often occurs among HUU children, the prevalence of stunting was higher among HUU children compared to HIV-exposed and HIV-free children. These findings point to the importance of prenatal efforts to reduce LBW and the consideration of modifiable postnatal factors to reduce stunting across all children, but particularly among HIV-exposed and HIV-free children, especially during the complementary feeding period.

LBW has been a persistent problem globally. A 2025 aim of the World Health Assembly’s Resolution 65.6, Comprehensive Implementation Plan on Maternal, Infant and Young Child Nutrition, focuses on strategies to reduce LBW [40]. To address the aim, a WHO-assembled global consultation of stakeholders recommended implementing multisectoral interventions that address maternal health, nutrition, psychosocial, environment, and socioeconomic domains prenatally as a strategy to improve maternal health and reduce the risk of LBW [41]. Findings from our study contribute evidence that reductions in LBW may reduce the risk of stunting among HIV-exposed and HIV-free children and provide suggestive evidence on the role of feeding practices, such as dietary diversity. The recommendations from the WHO stakeholders are consistent with the multisectoral interventions recommended by a Cochrane collaboration on preventing stunting [34].

In collaboration with UNICEF, the Deputy Prime Minister of the Kingdom of eSwatini is supporting a National Plan of Action for Children in eSwatini (2023–2027). The plan reviews the food and nutrition security policies as part of their initiative on child survival [42]. A recent analysis of food and nutrition security policies in eSwatini found that nutrition-specific programs included micronutrient supplementation, and deworming programs; breastfeeding campaigns; and treatment of acute malnutrition [43]. However, limited budgets, low coverage, and mismanagement of funds limited the impact of the program [43]. The review recommended a multisectoral coordinated approach that included the private sector and other stakeholders to establish dietary guidelines and collaborate in policy processes. Thus, multiple organizations have recognized that nutritional issues, such as stunting, are multisectoral and require action beyond nutrition-specific interventions.

### Strengths and Limitations

A major strength of our study is the availability of longitudinal stunting outcomes at 9 and 18 months that included repeated measures and multiple socio-demographic and caregiving variables, allowing us to study the trajectory of stunting in HIV-exposed and HIV-free children in eSwatini. In addition, we had data on some of the social determinants of health, including important child, maternal, and household characteristics that are known to have an effect on the growth and general well-being of infants [29,30]. This was a secondary analysis of data that were collected as part of an evaluation of Nurturing Care intervention effects on neurodevelopment in eSwatini. As such, the data were not intentionally powered to evaluate the effects of dietary and feeding practices, which may have impacted the ability to detect associations if they truly exist. Neither birth length nor the dietary variables at 18 months were available. In addition, the sample was drawn from clinics participating in a trial, which limits generalizability.

## 5. Conclusions

In this population of HIV-exposed and HIV-free children, stunting risk increased dramatically from 9 to 18 month of age, impacting over one-third of the children, consistent with the prevalence rate among a general population of children in eSwatini. The association with birthweight was strong, confirming that associations between birthweight and stunting found in HUU children also occurred in HIV-exposed and HIV-free children. The association between dietary diversity and stunting provides suggestive and novel evidence that further attention is needed to examine the social determinants of health, including feeding and dietary practices, that may modify the association between stunting and infant birthweight among HIV-exposed and HIV-free children. These findings also amplify the importance of the World Health Assembly’s Resolution 65.6 2025 aim to reduce low birthweight [40] and eSwatini’s National Plan of Action for Children in eSwatini [42]. As eSwatini increases the focus on survival, evidence from our study suggests that the prevention of both LBW and stunting can benefit the children of eSwatini, requiring a multisectoral plan with a prenatal component to promote maternal health and prevent LBW and a postnatal plan that incorporates the modifiable social determinants of health, such as dietary diversity, during the complementary feeding period. Reductions to the high stunting prevalence among children in eSwatini (greater than one-third), regardless of their HIV exposure, may improve children’s school performance and earnings in adult life, thereby enhancing human capital across the country.

## Figures and Tables

**Table 1:** Basic growth and dietary characteristics of the infants, mothers, and household at 9 and 18 months by stunting status.

	9 Months. N = 367			18 Months. N = 339		
Characteristic	Not Stunted	Stunted	*p*-Value	Not Stunted	Stunted	*p*-Value
	*n* = 291 *n* (%)	*n* = 76 *n* (%)		*n* = 216 *n* (%)	*n* = 123 *n* (%)	
Study arm			0.9			0.9
Intervention	149 (51.2)	38 (50.0)		112 (51.9)	65 (52.9)	
Comparison	142 (48.8)	38 (50.0)		104 (48.2)	58 (47.2)	
Child sex			0.52			0.575
Female	144 (49.48)	34 (44.74)		107 (49.54)	57 (46.34)	
Male	147 (50.52)	42 (55.26)		109 (50.46)	66 (53.66)	
Birthweight [median (IQR)]	3.2 (2.95–3.5)	3.0 (2.58–3.1)	<0.001	3.2 (3. 3.5)	3 (2.7. 3.25)	<0.001
Birthweight quartiles			0.001			<0.001
1.25–2.9	68 (23.37)	32 (42.11)		43 (19.91)	46 (37.4)	
2.95–3.1	54 (18.60)	23 (30.26)		37 (17.3)	32 (26.02)	
3.15–3.5	96 (33.00)	13 (17.11)		76 (35.19)	27 (21.95)	
3.57–5.0	61 (21.00)	4 (5.26)		49 (22.69)	13 (10.57)	
Missing	12 (4.12)	4 (5.26)		11 (5.09)	5 (4.07)	
Infant HIV-exposed, HIV-free nevirapine			0.442			0.341
No	193 (66.32)	46 (60.53)		143 (66.20)	72 (58.54)	
Yes	31 (10.65)	7 (9.21)		20 (9.26)	15 (12.20)	
Missing	67 (23.02)	23 (30.26)		53 (24.54)	36 (29.27)	
Dietary diversity score > 5			0.125			0.01
<5	128 (43.99)	41 (53.92)		83 (38.43	66 (53.66)	
=5	86 (29.55)	23 (30.26)		64 (29.63)	37 (30.08)	
>5	77 (26.46)	12 (15.79)		64 (29.63)	19 (15.45)	
Missing				5 (2.31)	1 (0.81)	
MinMealFreq score			0.25			0.71
No	55 (18.9)	15 (19.7)		39 (18.1)	23 (18.7)	
Yes	236 (81.1)	61 (80.3)		172 (79.6)	99 (80.5)	
Missing				5 (2.31)	1 (0.81)	
Responsive			1			0.3
No	265 (91.1)	69 (90.8)		197 (91.3)	109 (88.6)	
Yes	26 (8.9)	7 (9.2)		14 (6.5)	13 (10.6)	
Missing				5 (2.31)	1 (0.81)	
Controlling			0.17			0.12
No	242 (83.2)	60 (79)		182 (84.3)	94 (76.4)	
Yes	49 (16.8)	15 (19.7)		29 (13.4)	27 (21.9)	
Missing	0	1 (1.3)		5 (2.31)	1 (0.81)	
Indulgent			0.07			0.22
No	219 (75.3)	62 (81.6)		157 (72.7)	100 (81.3)	
Yes	72 (24.7)	13 (17.1)		54 (25)	21 (17.1)	
Missing	0	1 (1.3)		5 (2.31)	2 (1.63)	

**Table 2:** Multivariable regression for stunting at 9 months (Hypothesis 1).

	Odds Ratio	Lower 95% CI	Upper 95% CI	*p*-Value
Study Arm (ref is 1)	1.22	0.53	2.79	0.638
Exact age at 9-month evaluation (months)	0.96	0.71	1.30	0.784
Child gender (ref is female)				
Male	1.21	0.66	2.21	0.535
Residency (ref is rural)				
Periurban	0.79	0.31	2.01	0.618
Electricity (ref is none)	0.77	0.40	1.47	0.427
Clean water (ref is none)	0.72	0.38	1.37	0.313
Mother’s education group				
Std 1–5	2.38	0.79	7.17	0.123
Form 1–4	1.94	0.65	5.75	0.233
Form 5–Univ	0.75	0.19	3.01	0.688
Birthweight quartiles				
2.95–3.1	0.83	0.39	1.74	0.616
3.15–3.5	0.24	0.11	0.55	<0.001
3.57–5.0	0.10	0.03	0.33	<0.001
Dietary diversity score (ref is <5)				
5	0.86	0.44	1.71	0.675
>5	0.47	0.21	1.08	0.075
Minimum meal frequency (ref is no)	0.86	0.40	1.87	0.704
Feeding—controlling (ref is no) Yes	1.29	0.60	2.75	0.512
Feeding—indulgent (ref is no) Yes	0.72	0.34	1.55	0.405

**Table 3:** Multivariable regression for stunting at 9 months, testing interaction between birthweight and dietary diversity score (Hypothesis 2).

	Odds Ratio	Lower 95% CI	Upper 95% CI	*p*
Study arm (ref is 1)	1.21	0.55	2.70	0.634
Exact age at 9-month evaluation (months)	0.91	0.66	1.26	0.582
Child gender (ref is female)				
Male	1.32	0.71	2.43	0.377
Residency (ref is rural)				
Periurban	0.91	0.37	2.24	0.832
Electricity	0.80	0.41	1.58	0.525
Clean water (ref is none)	0.73	0.38	1.41	0.347
Mother’s education group				
Std 1–5	2.56	0.83	7.87	0.100
Form 1–4	1.93	0.64	5.79	0.242
Form 5–Univ	0.85	0.21	3.48	0.826
Birthweight quartiles				
2.95–3.1	2.02	0.75	5.49	0.167
3.15–3.5	0.29	0.09	0.95	0.040
3.57–5.0	0.22	0.05	0.97	0.046
Dietary diversity score (ref is <5)				
5	2.20	0.76	6.39	0.147
>5	0.77	0.21	2.80	0.693
Birthweight quartiles X dietary diversity categories				
2 2	0.07	0.01	0.51	0.009
2 3	0.29	0.04	2.06	0.215
3 2	0.65	0.12	3.59	0.625
3 3	0.72	0.09	6.12	0.766
4 2	* empty			
4 3	0.96	0.06	15.84	0.976
Feeding—controlling (ref is no) Yes	1.30	0.61	2.81	0.498
Feeding—indulgent (ref is no) Yes	0.77	0.35	1.69	0.521

* Not enough data points to generate estimates.

**Table 4:** Multivariable generalized structural equation model of association between birthweight and stunting at 18 months, mediated by stunting at 9 months.

Stunting at 18 Months	Odds Ratio	Lower 95% CI	Upper 95% CI	*p*-Value
Stunted at 9 months (ref is no)	23.80	9.10	62.26	<0.001
Birth weight quartiles				
2.95–3.1	0.87	0.29	2.56	0.797
3.15–3.5	0.50	0.20	1.25	0.139
3.57–5.0	0.34	0.16	0.75	0.007
Dietary diversity score categories (ref is <5)				
5	0.79	0.34	1.84	0.587
>5	0.41	0.18	0.91	0.029
Home observation score binary (ref is <16)	1.45	0.78	2.71	0.237
**Stunting at 9 months**				
Arm (ref = comparison)	1.28	0.58	2.82	0.535
Child gender (ref is female)				
Male	1.05	0.47	2.31	0.908
Residency (ref is rural)				
Periurban	0.91	0.41	2.03	0.819
Electricity	0.98	0.48	1.97	0.946
Water	0.65	0.43	1.00	0.049
Mother’s education group				
Std 1–5	3.24	1.09	9.63	0.035
Form 1–4	2.25	0.64	7.96	0.208
Form 5–Univ	1.07	0.25	4.56	0.928
Birthweight quartiles				
2.95–3.1				0.510
3.15–3.5	0.26	0.11	0.61	<0.001
3.57–5.0	0.11	0.04	0.34	<0.001
Dietary diversity score categories (ref is <5)				
5	0.83	0.43	1.59	0.568
>5	0.39	0.17	0.89	0.025
Feeding—controlling (ref is no) Yes	1.43	0.81	2.52	0.219
Feeding—indulgent (ref is no) Yes	0.67	0.34	1.31	0.246

## Data Availability

The original contributions presented in this study are included in the article/Supplementary Material. Further inquiries can be directed to the corresponding author(s).

## Supplementary Materials

The following supporting information can be downloaded at: https://www.mdpi.com/article/10.3390/nu18020198/s1, Supplemental Table S1. Complete characteristics of the infants, mothers and household at 9 and 18 months by stunting status; Supplementary Table S2: Total, direct and indirect effects of birth weight and diversity score on stunting at 18 months; Supplemental Table S3: Multivariable generalized structural equation model of association between birthweight and stunting at 18 months, mediated by stunting at 9 months, and interaction between birthweight and home observation score.

## Abbreviations

The following abbreviations are used in this manuscript:

adjOR	Adjusted odds ratio
ANC/PVT	Antenatal care/prevention of vertical transmission
DNA PCR	Deoxyribonucleic acid polymerase chain reaction
GLM	Generalized linear mixed models
HIV	Human immunodeficiency virus
HUU	HIV-unexposed and uninfected
HOME	Home observation for the measurement of the environment
IQR	Interquartile range
IYCF	Infant and young child feeding
Kg	Kilograms
LBW	Low birth weight
SES	Socioeconomic status
