# Supplementary material for: Stunting Among HIV-Exposed and HIV-Free Children in eSwatini: A Retrospective Evaluation of Associations with Birthweight, Feeding, and Caregiving Practices
Source: Nutrients. 2026 Jan 8;18(2):198. doi: 10.3390/nu18020198 (PMC12844829; doi:10.3390/nu18020198)
Supplement: Supplementary file 1 [file nutrients-18-00198-s001.zip › nutrients-4033280-supplementary.pdf]

Supplemental Table S1 Complete characteristics of the infants, mothers and household at 9 and 18 months by stunting status

Characteristic	9 - Months. N=367			18 - Months. N=333		
	Not Stunted n= 291	Stunted n= 76	P-value	Not Stunted n = 216	Stunted n =123	P-value
Study arm			0.9			0.9
Intervention	149(51.2)	38(50.0)		112(51.9)	65(52.9)	
Comparison	142(48.8)	38(50.0)		104(48.2)	58(47.2)	
Mother's Education (ref=none)			0.197			0.012
up to Gr2 + Std 1-5	111 (38.14)	34 (44.74)		77 (35.65)	55 (44.72)	
Form1-4	120 (41.24)	34 (44.74)		85 (39.35)	55 (44.72)	
Form5-Univ	55 (18.90)	7 (9.21)		46 (21.30)	11 (8.94)	
Missing	5 (1.72)	1 (1.32)		8 (3.70)	2 (1.63)	
Mother's Age Cat			0.826			0.294
18-24	77 (26.46)	20 (26.32)		52 (24.07)	36 (29.27)	
24-29	94 (32.30)	21 (27.63)		72 (33.33)	30 (24.39)	
30-34	75 (25.77)	23 (30.26)		57 (26.39)	33 (26.83)	
35+	45 (15.46)	12 (15.79)		30 (13.89)	23 (18.70)	
Missing				5 (2.31)	1 (0.81)	
Child sex			0.52			0.575
Female	144 (49.48)	34 (44.74)		107 (49.54)	57 (46.34)	
Male	147 (50.52)	42 (55.26)		109 (50.46)	66 (53.66)	
SES			**0.026**			**0.023**
Most poor	67 (23.02)	25 (32.89)		49 (22.69)	36 (29.27)	
Least poor	63 (21.65)	23 (30.26)		42 (19.44)	36 (29.27)	
Middle	87 (29.90)	15 (19.74)		60 (27.78)	26 (21.14)	
Least poor	57 (19.59)	7 (9.21)		48 (22.22)	14 (11.38)	
Missing	17 (5.84)	6 (7.89)		17 (7.87)	11 (8.94)	
Residency			0.176			0.259
Rural	185 (63.57)	55 (72.37)		133 (61.57)	86 (69.92)	
Peri urban	106 (36.43)	21 (27.63)		78 (72.6)	36 (29.27)	
Missing				5 (2.31)	1 (0.81)	
Electricity			0.077			**0.041**
No	126 (43.30)	44 (57.89)		89 (41.20)	67 (54.47)	
Yes	163 (56.01)	32 (42.11)		121 (56.02)	55 (44.72)	
Missing	2 (0.69)	0 (0.00)		6 (2.78)	1 (0.81)	

Clean water			0.011			0.223
No	139 (47.77)	50 (65.79)		103 (47.69)	70 (56.91)	
Yes	151 (51.89)	26 (34.21)		108(50.00)	52 (42.28)	
Missing	1 (0.34)	0		5 (2.31)	1 (0.81)	
Toilet			0.08			0.068
No	272 (93.47)	76 (100)		197 (91.20)	120 (97.56)	
Yes	17 (5.84)	0 (0.00)		13 (6.02)	2 (1.63)	
Missing	2 (0.69)	0 (0.00)		6 (2.78)	1 (0.81)	
Mother's Marital status			0.971			0.918
Never Married	89 (30.58)	23 (30.26)		67 (31.02)	36 (29.27)	
Currently Married	145 (49.83)	37 (48.68)		104(48.15)	63 (51.22)	
Other	52 (17.87)	15 (19.74)		37 (17.13)	21 (17.07)	
Missing	5 (1.72)	1 (1.32)		8 (3.70)	3 (2.44)	
Mother Employment status			0.261			0.516
Employed	83 (28.52)	27 (35.53)		60 (27.78)	39 (31.71)	
Unemployed	208 (71.48)	49 (64.47)		151(69.91)	83 (67.48)	
Missing				5 (2.31)	1 (0.81)	
Maternal depression			0.693			0.635
EPDS <13	177 (60.82)	44 (57.89)		130 (60.19)	73 (59.35)	
EPDS >= 13	114 (39.18)	32 (42.11)		81 (37.50)	49 (39.84)	
Missing				5 (2.31)	1 (0.81)	
Birth weight [Median (IQR)]	3.2 (2.95-3.5)	3.0 (2.58-3.1)	<0.001	3.2 (3. 3.5)	3 (2.7. 3.25)	<0.001
Birth weight quartiles			0.001			<0.001
1.25 - 2.9	68(23.37)	32(42.11)		43(19.91)	46(37.4)	
2.95 - 3.1	54(18.60)	23(30.26)		37(17.3)	32(26.02)	
3.15 - 3.5	96(33.00)	13(17.11)		76(35.19)	27(21.95)	
3.57 - 5.0	61(21.00)	4(5.26)		49(22.69)	13(10.57)	
Missing	12 (4.12)	4 (5.26)		11 (5.09)	5 (4.07)	
Timing of ART start (maternal)			0.613			0.645
Before Preg	154 (52.92)	39 (51.32)		106 (49.07)	66 (53.66)	
During Preg	116 (39.86)	29 (38.16)		92 (42.59)	46 (37.40)	
Missing	21 (7.22)	8 (10.53)		18 (8.33)	11 (8.94)	
Infant HEU nevirapine			0.442			0.341
No	193 (66.32)	46 (60.53)		143 (66.20)	72 (58.54)	
Yes	31 (10.65)	7 (9.21)		20 (9.26)	15 (12.20)	
Missing	67 (23.02)	23 (30.26)		53 (24.54)	36 (29.27)	

Home inventory score binary	Not applicable					0.917
<16				114 (52.78)	64 (52.03)	
16				101 (46.76)	58 (47.15)	
				1 (0.46)	1 (0.81)	
Dietary diversity score >5			0.125			0.01
<5	128(43.99)	41(53.92)		83(38.43	66(53.66)	
`=5	86(29.55)	23(30.26)		64(29.63)	37(30.08)	
>5	77(26.46)	12(15.79)		64(29.63)	19(15.45)	
Missing				5 (2.31)	1 (0.81)	
MinMealFreq score			0.25			0.71
No	55(18.9)	15(19.7)		39(18.1)	23(18.7)	
Yes	236(81.1)	61(80.3)		172(79.6)	99(80.5)	
Missing				5(2.31)	1 (0.81)	
Responsive			1			0.3
No	265(91.1)	69(90.8)		197(91.3)	109(88.6)	
Yes	26(8.9)	7(9.2)		14(6.5)	13(10.6)	
Missing				5(2.31)	1 (0.81)	
Controlling			0.17			0.12
No	242(83.2)	60(79)		182(84.3)	94(76.4)	
Yes	49(16.8)	15(19.7)		29(13.4)	27(21.9)	
Missing	0	1(1.3)		5(2.31)	1 (0.81)	
Indulgent			0.07			0.22
No	219(75.3)	62(81.6)		157(72.7)	100(81.3)	
Yes	72(24.7)	13(17.1)		54(25)	21(17.1)	
Missing	0	1(1.3)		5(2.31)	2(1.63)	

Supplemental Table S2: Total, direct and indirect effects of birth weight and diversity score on stunting at 18 months

		Total effect			Direct effect			Indirect effect		
		Coeff	Lower CI	Upper CI	Coeff	Lower CI	Upper CI	Coeff	Lower CI	Upper CI
Birth weight quartile	2	-0.80	-3.02	1.42	-0.14	-1.22	0.94	-0.66	-2.58	1.27
	3	-5.00**	-7.51	-2.50	-0.69	-1.60	0.22	-4.32*	-7.14	-1.50
	4	-7.97*	-12.57	-3.38	-1.06*	-1.84	-0.29	-6.91*	-11.61	-2.21
Diversity score	2	-0.84	-2.98	1.30	-0.23	-1.08	0.61	-0.60	-2.61	1.40
	3	-3.86*	-6.56	-1.15	-0.90*	-1.71	-0.09	-2.96*	-5.76	-0.15
**p-value<0.001<br>*p-value<0.05										

Supplemental Table S3: Multivariable generalized structural equation model of association between birthweight and stunting at 18 months, mediated by stunting at 9 months; and interaction between birthweight and home observation score

Part 1: Stunting at 18 months	Odds Ratio	Lower 95% CI	Upper 95% CI	p-value
Stunted at 9 month(ref is no)	24.07	9.28	62.45	<0.001
Birth weight quartiles				
2.95 - 3.1	0.95	0.22	4.11	0.949
3.15 - 3.5	0.44	0.11	1.72	0.238
3.57 - 5.0	0.40	0.11	1.50	0.176
Home Observation Score Binary (ref is <16)	1.48	0.44	4.93	0.527
birth weight quartiles X home observation score				
2 1	0.82	0.19	3.46	0.786
3 1	1.30	0.33	5.07	0.709
4 1	0.74	0.13	4.30	0.733

Dietary diversity score categories (ref is <5)				
5	0.81	0.35	1.88	0.626
>5	0.41	0.18	0.90	*0.027*
**Part 2: Stunting at 9 months**				
Arm (ref is 1)	1.28	0.58	2.82	0.535
Baby Gender (ref is female)				
M	1.05	0.47	2.31	0.908
Residency (ref is rural)				
Peri-urban	0.91	0.41	2.03	0.819
Electricity	0.98	0.48	1.97	0.946
Water	0.65	0.43	1.00	0.049
Mom Edu 3 groups				
Std 1-5	3.24	1.09	9.63	0.035
Form1-4	2.25	0.64	7.96	0.208
Form5-Univ	1.07	0.25	4.56	0.928
Birth weight quartiles				
2.95 - 3.1	0.81	0.44	1.51	0.510
3.15 - 3.5	0.26	0.11	0.61	0.002
3.57 - 5.0	0.11	0.04	0.34	<0.001
Dietary diversity score categories (ref is <5)				
5	0.83	0.43	1.59	0.568
>5	0.39	0.17	0.89	0.025
Feeding - Controlling (ref is no)				0.219
Yes	1.43	0.81	2.52	
Feeding- Indulgent (ref is no)				0.246
Yes	0.67	0.34	1.31	
